# Preoperative plasma fibrinogen combined with the platelet-to-lymphocyte ratio (F-PLR) serves as a prognostic indicator in patients with non-small cell lung cancer

**DOI:** 10.3389/fonc.2025.1587443

**Published:** 2025-10-31

**Authors:** Hongzhen Zhao, Xiaopeng Zhang, Dahu Ren, Shicheng Liu, Yuedong Wang, Wenbo Wu, Kun Zhang, Yajing Niu, Guochen Duan

**Affiliations:** ^1^ Graduate School, Hebei Medical University, Shijiazhuang, China; ^2^ Department of Thoracic Surgery, Hebei General Hospital, Shijiazhuang, China; ^3^ Department of Cardiovascular Surgery, Hebei General Hospital, Shijiazhuang, China; ^4^ Department of Geriatric Cardiovascular Medicine, Hebei General Hospital, Shijiazhuang, China

**Keywords:** F-PLR, NSCLC, prognostic, inflammatory response, coagulation system

## Abstract

**Background:**

The aim of this study was to explore the prognostic significance of the combined plasma fibrinogen level and platelet-to-lymphocyte ratio (F-PLR) score in patients who had undergone radical surgery for non-small cell lung cancer (NSCLC).

**Methods:**

In this study, we retrospectively reviewed the medical records of 214 patients who underwent radical resection for lung cancer. The optimal cut-off values for fibrinogen and the platelet - lymphocyte ratio (PLR) were determined by applying the receiver operating characteristic (ROC) curve and the Youden index. Based on these cut-off values, the patients were categorized into three groups: patients with elevated fibrinogen and PLR were assigned a score of 2; those with either elevated fibrinogen or PLR were assigned a score of 1; and those with neither elevation were assigned a score of 0. The Kaplan-Meier method was utilized to plot the survival curves, and differences among the curves were compared using the log - rank test. Univariate and multivariate analyses were carried out using the Cox proportional hazards model.

**Results:**

In this study, the optimal cutoff values were 3.90 for fibrinogen and 213.2 for the PLR. Cox’s multifactorial analysis revealed that the implementation of adjuvant therapy after surgery(P<0.001), pathological stage(PStage=IIIA/I=0.041), and F-PLR(PF-PLR=1/0 = 0.006、PF-PLR=2/0 = 0.004)were independent prognostic factors influencing patient survival. Additionally, F-PLR was significantly correlated with the overall survival of NSCLC patients after surgery.

**Conclusions:**

The F-PLR score exhibits a significant association with the prognosis of NSCLC patients and can serve as a biomarker for predicting the prognosis of patients following NSCLC surgery.

## Introduction

Based on the latest 2024 statistics, lung cancer persists in being the malignancy with the highest global incidence and mortality rates. Non-small cell lung cancer (NSCLC) is the predominant histological subtype of lung cancer worldwide, accounting for a large proportion of all diagnosed cases (1). In recent years, remarkable advancements have been made in multimodal tumor treatment. Nevertheless, surgical treatment remains the cornerstone for early stage NSCLC. Regrettably, the enhancement of patients’ postoperative survival outcomes leaves much to be desired. Specifically, the median postoperative survival of Chinese NSCLC patients is merely 22.7 months (2). Consequently, a subset of patients frequently necessitate proactive postoperative adjuvant therapeutic interventions, including chemotherapy and immunotherapy (3, 4). Furthermore, even among patients with identical staging who have received aggressive postoperative adjuvant therapies, substantial disparities in postoperative survival times still persist (5). Identifying a reliable prognostic indicator is of paramount importance. This enables clinicians to stratify the risk levels of patients who have undergone lung cancer surgery and subsequently customize the treatment plans for them.

The systemic inflammatory response and the activation of the coagulation system exert crucial influences on tumor proliferation, metastasis, and tumor-associated angiogenesis. These processes are intimately associated with the poor prognosis of tumor patients (6, 7). The cancer-related systemic inflammatory milieu, quantifiable through hematologic indices including the neutrophil-to lymphocyte ratio (NLR) and the platelet-to-lymphocyte ratio (PLR), has emerged as a key pathophysiological determinant of oncologic outcomes, demonstrating consistent prognostic validity across multiple thoracic oncology cohorts (8, 9). Plasma fibrinogen, a key coagulation-associated protein, plays a significant role in tumor cell proliferation, metastasis, and tumor-associated angiogenesis by directly binding to tumor cells (10). Elevated plasma fibrinogen levels are considered to be correlated with poor prognosis in patients with ovarian cancer, cholangiocarcinoma, colorectal cancer, and non-small cell lung cancer (11–14).

However, when single indicators are applied to predict the prognosis of tumor patients, they often fail to achieve satisfactory predictive efficacy. Therefore, the combination of blood-based biomarkers has been receiving increasing emphasis and demonstrates promising application prospects. Several recent studies have evaluated plasma fibrinogen levels and the neutrophil-to-lymphocyte ratio (F-NLR),the lymphocyte-to-monocyte ratio (F-LMR), and the serum albumin level (FA) has been utilized to stratify the prognostic risk for gastric, colorectal, and NSCLC and has demonstrated robust predictive ability (15–17). Huang J et al. (18) employed the preoperative F-PLR score to assess the prognosis of patients with limited upper uroepithelial carcinoma. The results indicated that the higher the F-PLR score, the poorer the patient’s prognosis, and it served as an independent prognostic factor for these patients. Yang Y et al. (19) used the F-PLR score to evaluate the prognosis of esophageal squamous carcinoma. The outcomes showed that compared to esophageal squamous carcinoma patients with low F-PLR scores, those with high F-PLR scores experienced significantly shorter overall survival (OS) and disease-free survival (DFS). Additionally, the study demonstrated that high F-PLR scores were significantly correlated with tumor length and infiltration depth. The F-PLR score encompasses clinical markers reflecting the body’s fibrinolytic status, coagulation function, and inflammatory response, demonstrating greater clinical application value compared to indicators solely representing inflammatory or coagulation functions. To our knowledge, no research has been published on applying the F-PLR score for postoperative prognostic risk assessment in NSCLC patients. Therefore, we initiated a clinical study to explore whether the F-PLR scoring system could be utilized to evaluate the postoperative prognosis of NSCLC patients.

## Materials and methods

This study was carried out in strict accordance with the principles of the Declaration of Helsinki. The retrospective study was approved by the Ethics Committee of Hebei General Hospital and patients’ informed consent was exempted(2025-LW-0045).

### Patients

This study recruited 486 patients who underwent radical resection for lung cancer at the Thoracic Surgery Department of Hebei General Hospital between January 2020 and December 2021. The inclusion criteria for the cases were as follows:

1. The patients were pathologically confirmed to have NSCLC post-operation.
2. Radical lung cancer resection and systematic lymph node dissection were carried out.

The criteria employed for exclusion were as listed below:

1. Patients who underwent neoadjuvant therapy before the surgical procedure.
2. Patients lacking relevant test results, such as plasma fibrinogen levels, platelet and lymphocyte counts, and those with incomplete medical records before surgery.
3. Patients with a history of acute infections (defined as body temperature >38 °C, or C-reactive protein [CRP] level >10 mg/L, or white blood cell [WBC] count >10×10^9^/L within one week before surgery), hematological disorders (including but not limited to leukemia, lymphoma, myelodysplastic syndromes, hemophilia, etc.), and autoimmune diseases (including but not limited to rheumatoid arthritis, systemic lupus erythematosus, etc.).
4. Patients with primary tumors in other sites, where the lung lesion was a metastatic site.

Based on the above criteria, a total of 214 patients were enrolled in the study. Among these patients, 111 were male and 103 were female. Regarding postoperative pathological diagnoses, 178 patients were diagnosed with lung adenocarcinoma,28 with squamous-cell lung cancer, and 8 with other tumor types.

### Data collection and follow-up

Demographic characteristics, along with relevant test and examination results upon admission, were retrieved from the hospital medical record management system. These included age, gender, smoking status, and relevant data such as platelet count, lymphocyte count, fibrinogen level, and tumor-lymph node-metastasis (TNM) staging. The TNM staging was determined according to the 8th edition of the guidelines issued by the International Association for the Study of Lung Cancer. For adjuvant treatment regimens, patients with lung squamous cell carcinoma received platinum-based agents plus albumin-bound paclitaxel with or without immunotherapy; those with lung adenocarcinoma were treated with platinum-based agents plus pemetrexed with or without immunotherapy and/or targeted therapy; while adjuvant regimens for other pathological types of lung cancer strictly adhered to the Chinese Society of Clinical Oncology (CSCO) Clinical Guidelines for Primary Lung Cancer (2019 edition).

In the first year following surgery, follow-up evaluations were carried out every 3 months. In the second postoperative year, the follow-up interval was set at every 6 months. Starting from the third postoperative year, follow-up was conducted once a year. The final follow-up was completed on February 2025. Overall survival (OS) was defined as the time from the date of surgery to the date of death from any cause. For patients who were still alive at the last follow-up or were lost to follow-up, their data were censored on the last date when they were known to be alive.

### F-PLR

Blood was drawn from all patients within three days prior to surgery for routine blood tests, biochemical examinations, and coagulation function examinations. The PLR values were calculated based on the obtained results. The calculation formula was PLR=peripheral blood platelet count(10^9^/L)/peripheral blood lymphocyte count (10^9^/L).

### Statistical analysis

Data analysis was carried out with the help of SPSS 26.0 statistical software. The receiver operating characteristic (ROC) curve was plotted to assess the sensitivity and specificity of overall survival. By calculating Youden’s index, the optimal cut-off values for the PLR and fibrinogen were identified. The chi-square test was employed to analyze the associations between the three F-PLR score groups and the clinical baseline data. Survival curves for the three groups were estimated using the Kaplan-Meier method and compared by a log-rank test. Internal validation was performed using 1000 bootstrap samples to calculate the optimism-corrected area under the curve (AUC) and assess potential overfitting risk. The Cox proportional-hazards model was utilized to calculate the hazard ratio (HR) and 95% confidence interval (CI) through univariate and multivariate analyses. Variables that showed statistical significance in the univariate analysis were included in the multivariate analysis. P <0.05 was taken to indicate statistical significance.

**Figure 1:**
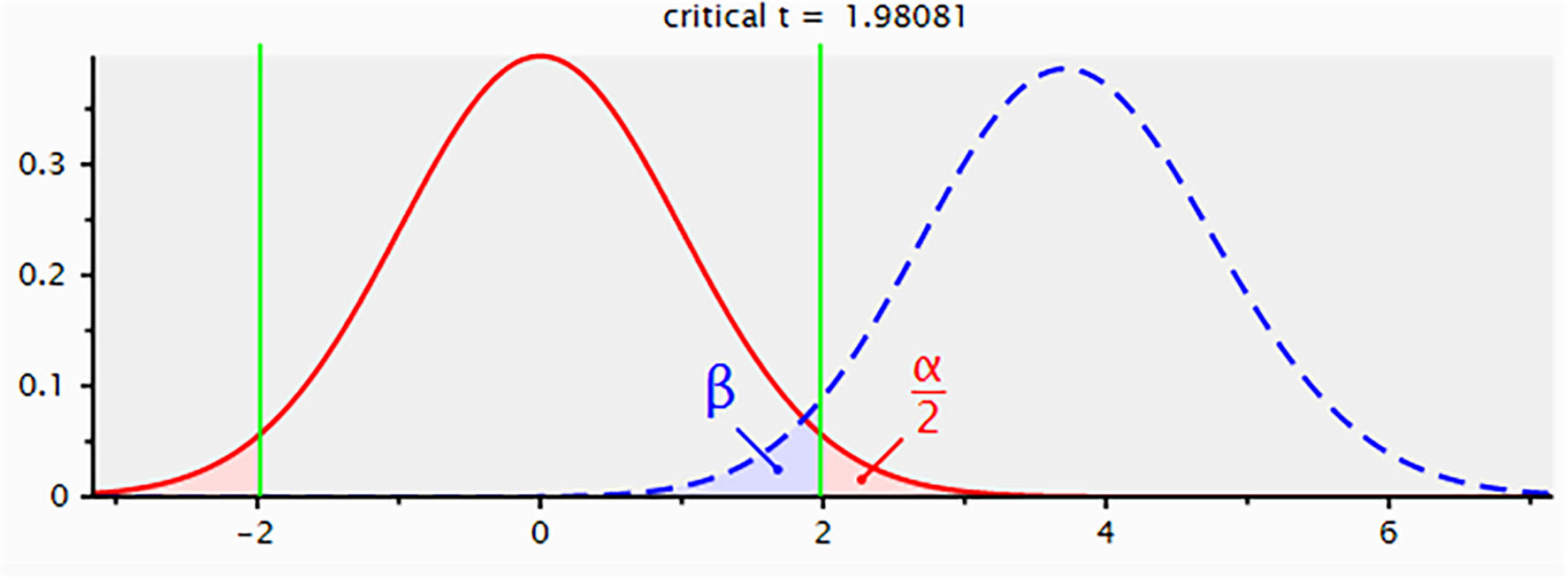
*Post hoc* power analysis for the comparison of survival between F-PLR groups.

We performed a *post hoc* power analysis using G Power software (version 3.1.9.2) to validate the adequacy of our sample size. The analysis modeled the substantial survival difference between the F-PLR=0 (n=88) and F-PLR=2 (n=29) groups as a large effect size (Cohen’s d=0.8) and estimated the statistical power through an independent samples t-test. The results demonstrated a statistical power of 96.0%, confirming that our study sample size provided sufficient power to detect significant prognostic effects (**Figure 1**).

Due to the strict patient selection criteria that excluded individuals with incomplete medical records or missing key laboratory data, the analytical dataset for the 214 enrolled patients was complete, and no data imputation was necessary.

## Results

### Characteristics of patients

**Table 1** presents the clinical baseline data of patients who were classified into three groups according to the F-PLR score. In the present study, a total of 214 patients were enrolled. Among these patients, 111 (51.9%) were male and 103 (48.1%) were female. The patients included in this study were aged from 33 to 83 years old, with 99 (47.3%) of the patients aged ≤60 years and 115 (53.7%) of the patients aged >60 years. In accordance with the TNM staging guidelines of the 8th edition issued by the International Association for the Study of Lung Cancer, 125 patients were categorized as stage I, 58 as stage II, and 31 as stage IIIA. Based on the F-PLR score, the patients were classified into three groups: 88 patients had an F-PLR value of 0, 97 patients had an F-PLR value of 1, and 29 patients had an F-PLR value of 2.

**Table 1:** The basic patient information and the relationships among the three groups of F-PLR patients.

Variables	F-PLR=0 n (%)	F-PLR=1 n (%)	F-PLR=2 n (%)	P value
Age (year)				0.003
≤60	42 (47.7%)	36 (37.1%)	21 (72.4%)	
>60	46 (52.3%)	61 (62.9%)	8 (27.6%)	
Sex				0.789
Female	40 (45.5%)	49 (50.5%)	14 (48.3%)	
Male	48 (54.5%)	48 (49.5%)	15 (51.7%)	
Smoking				0.172
Yes	35 (39.8%)	26 (26.8%)	10 (34.5%)	
No	53 (60.2%)	71 (73.2%)	19 (65.5%)	
Tumor location				0.602
Upper lobe of the right lung	36 (40.9%)	33 (34.0%)	7 (24.1%)	
Lower lobe of the right lung	14 (15.9%)	14 (14.4%)	7 (24.1%)	
Middle lobe of the right lung	4 (4.5%)	10 (10.3%)	2 (6.9%)	
Upper lobe of the left lung	16 (18.2%)	19 (19.6%)	8 (27.6%)	
Lower lobe of the left lung	18 (20.5%)	21 (21.6%)	5 (17.2%)	
Histological subtype				0.135
Adenocarcinoma	76 (86.4%)	82 (84.5%)	20 (69.0%)	
SqCC	10 (11.4%)	10 (10.3%)	8 (27.6%)	
Others	2 (2.3%)	5 (5.2%)	1 (3.4%)	
Resection type				0.452
Wedge resection	17 (19.3%)	17 (17.5%)	5 (17.2%)	
Segmental lung resection	9 (10.2%)	3 (3.1%)	3 (10.3%)	
Lobectomy	61 (69.3%)	76 (78.4%)	21 (72.5%)	
Sleeve resection	1 (1.2%)	1 (1.0%)	0 (0.0%)	
Pathological stage				0.042
I	58 (65.9%)	55 (56.7%)	12 (41.4%)	
II	20 (22.7%)	30 (30.9%)	8 (27.6%)	
IIIA	10 (11.4%)	12 (12.4%)	9 (31.0%)	

F-PLR, plasma fibrinogen level and platelet-to-lymphocyte ratio; SqCC, squamous cell carcinoma.

### Relationship between F-PLR and clinicopathological

The associations between F-PLR and the patients’ baseline data are presented in **Table 1**. Among these, F-PLR was associated with age(P=0.003)and the patients’ pathological stage(P=0.042), while it was not associated with the patients’ gender(P=0.789), smoking history(P=0.172), tumor location(P=0.602), tumor pathology type(P=0.135), and surgical approach(P=0.452).

### Receiver operating characteristic curve for overall-survival prediction

The optimal cut-off values for the patients’ preoperative fibrinogen levels and PLR were determined from the ROC curves. For fibrinogen, the optimal cut-off value was 3.90 g/L, with a sensitivity of 58.6%, a specificity of 87.6%, and an area under the ROC curve (AUC) of 0.741(95%CI:0.642-0.840).Regarding the F-PLR, the optimal cut-off value was determined to be 213.2. The sensitivity was 91.9%, the specificity was 55.9%, and the area under the AUC was 0.753(95%CI:0.681-0.826).

**Figure 2:**
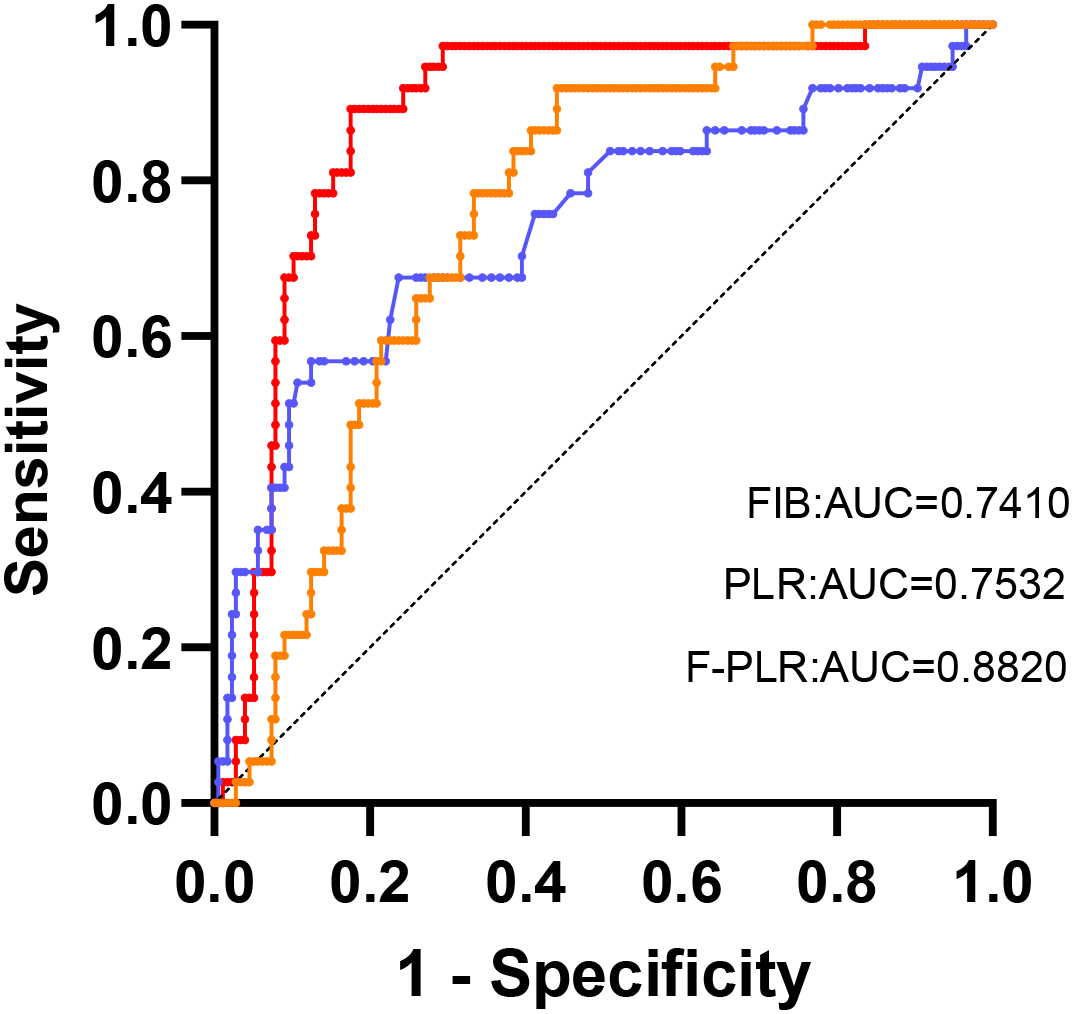
The ROC analysis was utilized to evaluate the predictive accuracy of OS and to ascertain the optimal cutoff values for FIB and the PLR.

The ROC curves constructed based on the OS of patients demonstrated AUCs of 0.741, 0.753, and 0.882 for fibrinogen, PLR, and F-PLR, respectively (**Figure 2**). Internal validation using 1000 bootstrap resamples demonstrated an optimism-corrected AUC of 0.812 (95% CI: 0.759-0.857) for F-PLR, confirming the robustness of its predictive performance. The results indicated that, in NSCLC patients who had undergone surgical treatment, the prognostic predictive ability of F-PLR surpassed that of fibrinogen and PLR when used independently.

### Prognostic analysis based on F-PLR among patients with NSCLC

**Figure 3:**
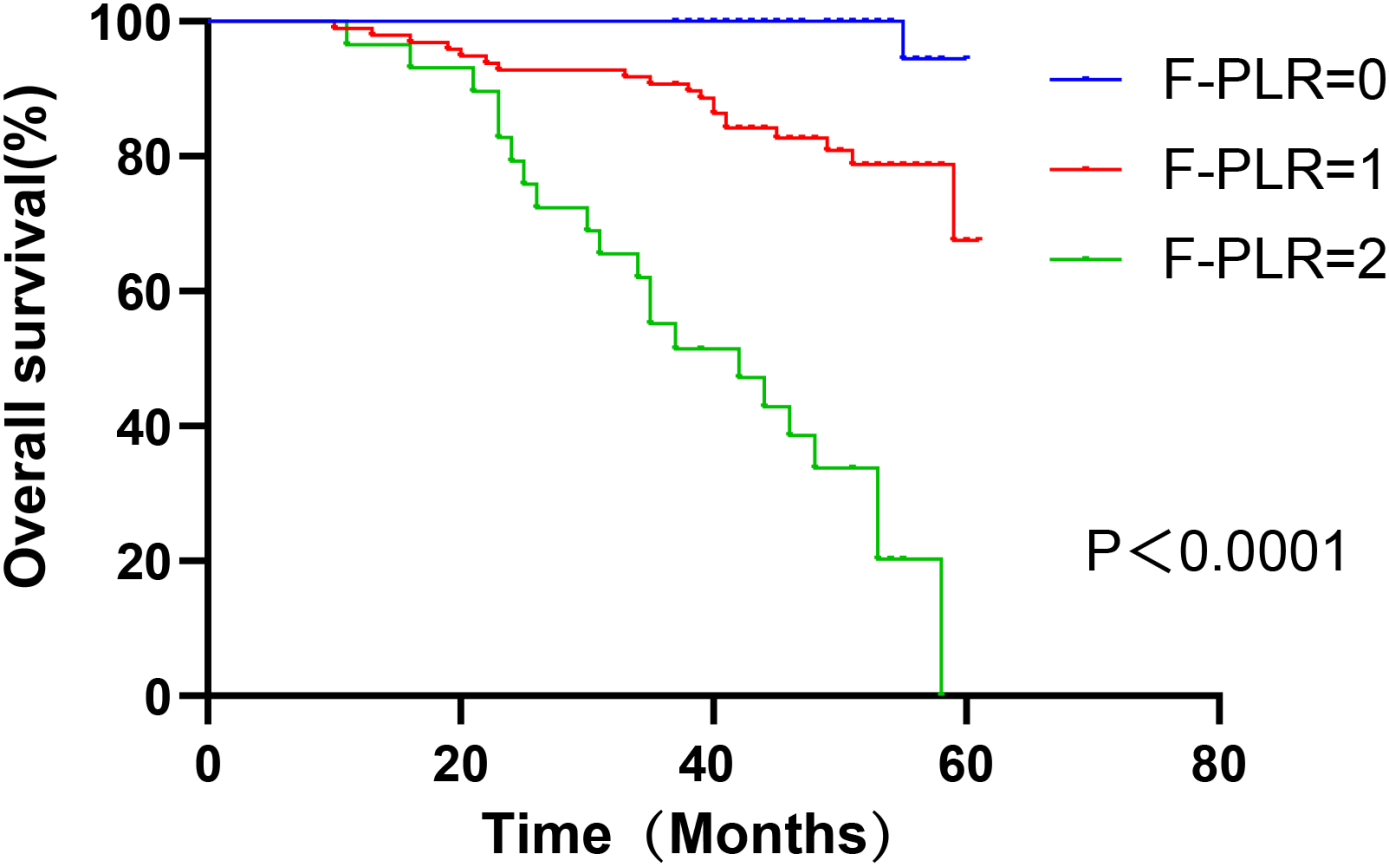
Kaplan-Meier curves for NSCLC patients according to F-PLR score.

**Figure 4:**
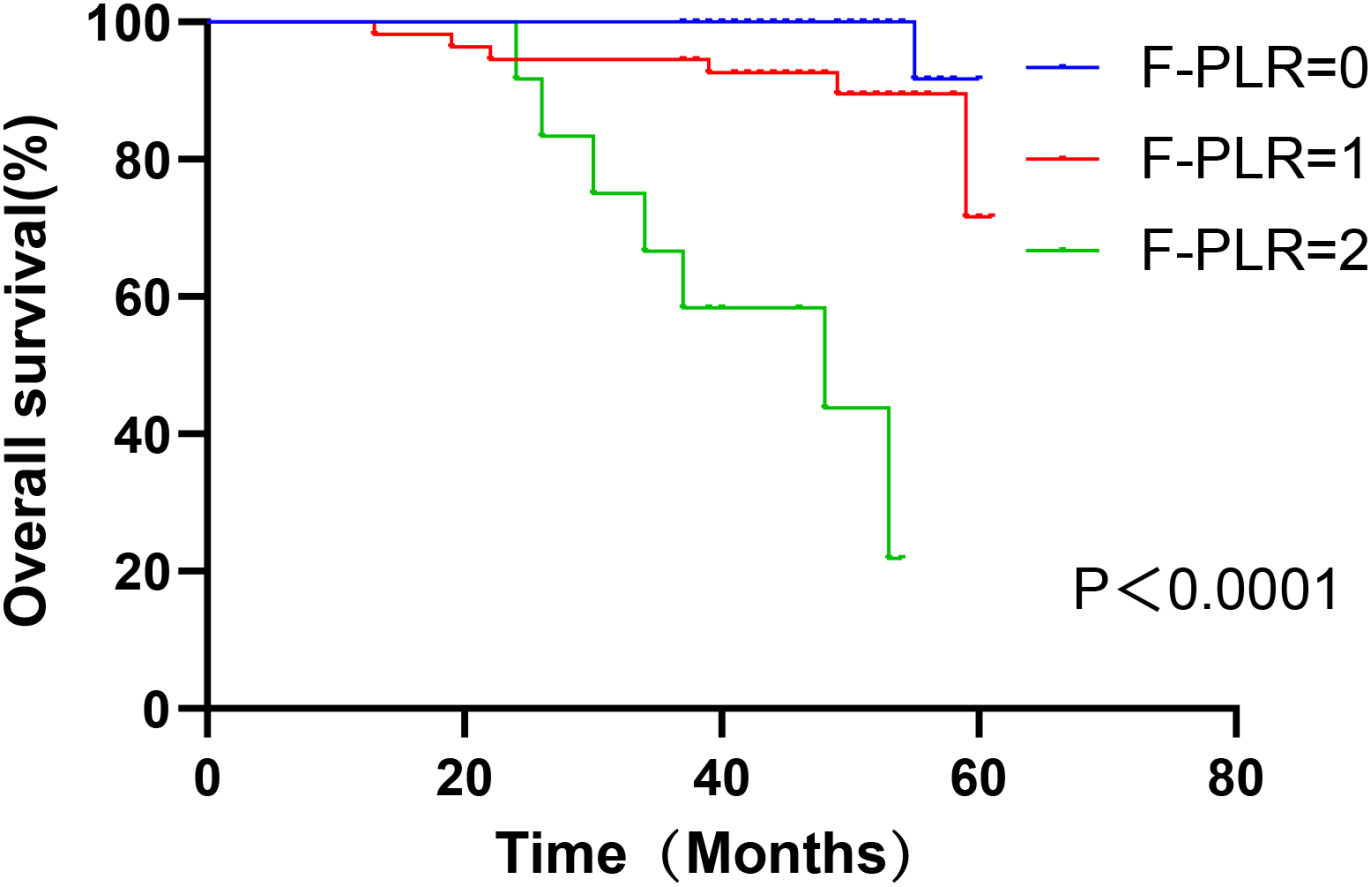
Kaplan-Meier curves of stage I NSCLC patients according to F-PLR score.

**Figure 5:**
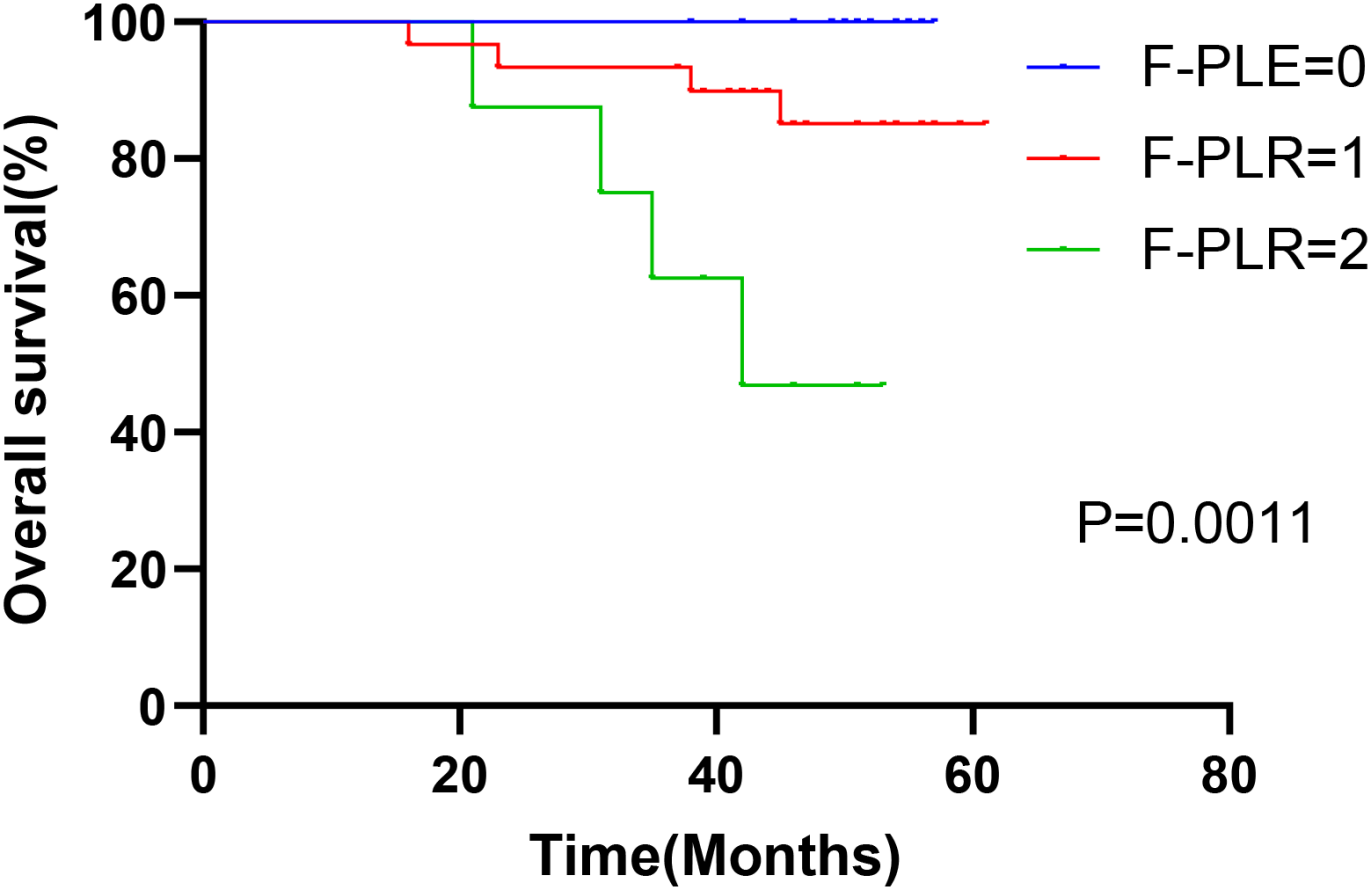
Kaplan-Meier curves of stage II NSCLC patients according to F-PLR score.

**Figure 6:**
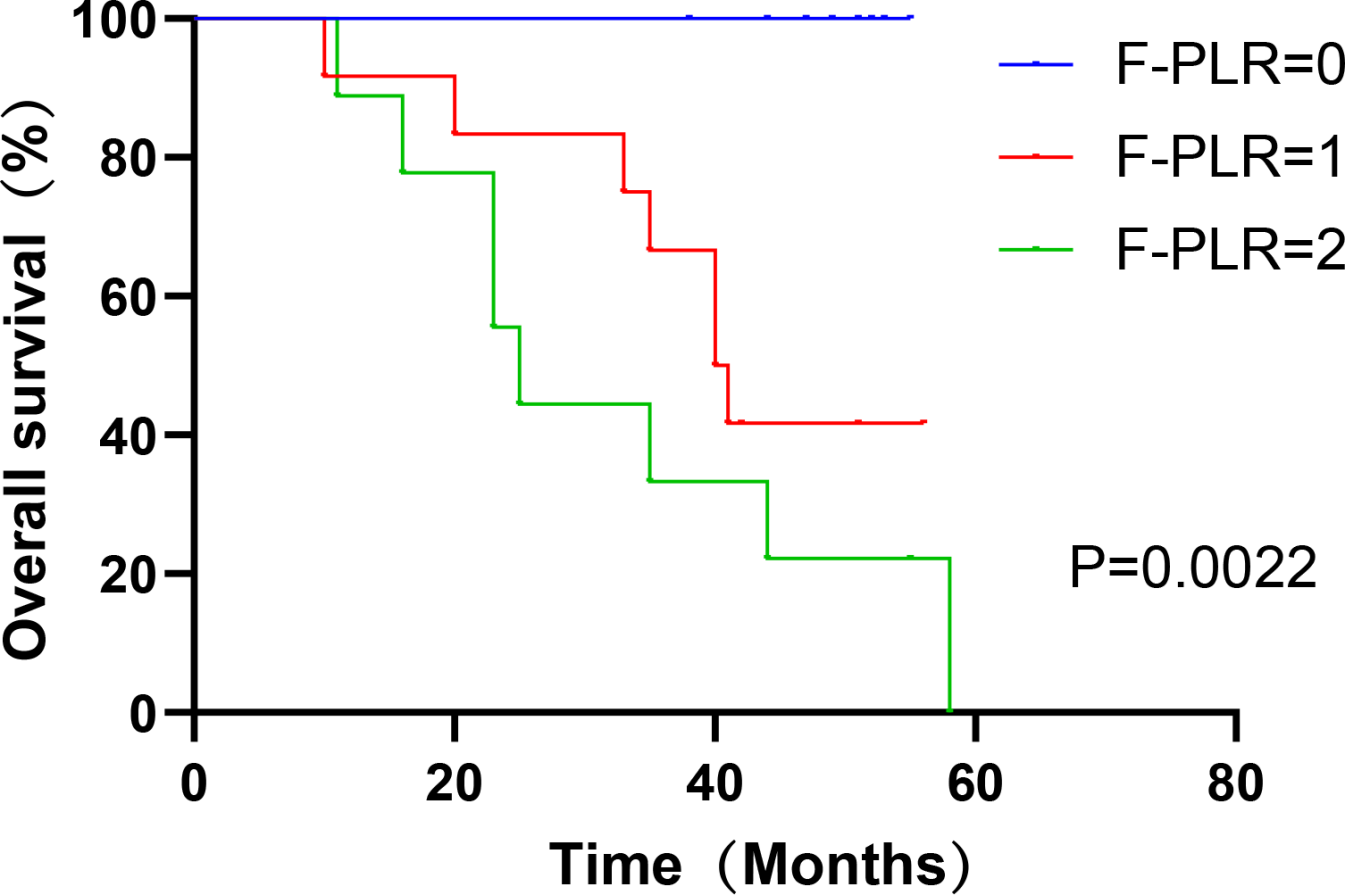
Kaplan-Meier curves of stage IIIA NSCLC patients according to F-PLR score.

In surgically treated NSCLC patients, F-PLR was significantly associated with the OS(PF-PLR=1/0 = 0.006、PF-PLR=2/0 = 0.004) of the patients (**Table 2**). The 1-year, 3-year, and 5-year survival rates in the F-PLR = 0 group were 100%, 100%, and 94.4%, respectively. In the F-PLR = 1 group, these rates were 97.94%, 90.72%, and 71.69%, respectively. For the F-PLR = 2 group, the 1-year, 3-year, and 5-year survival rates were 93.10%, 51.72%, and 0.00%, respectively (**Figure 3**). Subsequently, we separately analyzed the OS of patients at different pathological stages. The analysis results indicated that, in patients with stages I, II, and III, the survival rate of those with F-PLR=0 was higher than that of the patient populations with F-PLR=1 and F-PLR=2, and the differences were statistically significant(P<0.0001, P=0.0011, P=0.0022) (**Figures 4**–**6**).

**Table 2:** Univariate and multifactorial analyses of OS among three groups of patients.

Variables	Univariate		Multivariate	
	HR (95% CI)	P value	HR (95% CI)	P value
Age (years)				
≤60	Reference			
>60	1.40 (0.73-2.71)	0.314		
Sex				
Female	Reference		Reference	
Male	1.93 (1.02-3.65)	0.044	1.67 (0.85-3.28)	0.139
Adjuvant therapy				
Yes	Reference		Reference	
No	8.94 (4.55-17.57)	<0.001	6.09 (2.85-13.01)	<0.001
Pathological stage				
I	Reference		Reference	
II	1.47 (0.66-3.27)	0.347	1.21 (0.53-2.79)	0.648
IIIA	5.87 (2.88-11.94)	<0.001	2.29 (1.03-5.07)	0.041
Surgical				
Wedge resection	Reference			
Segmental lung resection	1.37 (0.25-7.47)	0.718		
Lobectomy	2.31 (0.82-6.54)	0.113		
Sleeve resection	7.01 (0.78-63.31)	0.083		
FIB				
≤3.90	Reference		Reference	
>3.90	6.94 (3.70-13.00)	<0.001	0.43 (0.06-3.16)	0.410
PLR				
≤213.2	Reference		Reference	
>213.2	9.54 (3.39-26.83)	<0.001	0.56 (0.11-2.90)	0.487
F-PLR score				
0	Reference		Reference	
1	17.69 (2.36-13.50)	0.005	36.89 (2.82-8.99)	0.006
2	100.477 (13.48-14.81)	<0.001	240.49 (5.50-10.69)	0.004

OS, overall survival; FIB, Fibrinogen; F-PLR, plasma fibrinogen level and platelet-to-lymphocyte ratio; HR, hazard ratio; CI, confidence interval.

### Univariate and multivariate assessments of OS

The results of the univariate analysis of patients’ OS indicated that gender(P=0.044), the adoption of adjuvant therapy after surgery(P<0.001), pathological stage(PStage=IIIA/I<0.001), fibrinogen level(P<0.001), PLR(P<0.001), and F-PLR(PF-PLR=1/0 = 0.005、PF-PLR=2/0<0.001)were associated with patients’ survival. The results of the subsequent multivariate analysis indicated that the utilization of adjuvant therapy after surgery(P<0.001, HR=6.09, 95% CI=2.85-13.01), pathological staging(PStage=IIIA/I=0.041,HR=2.29, 95% CI=1.03-5.07), and F-PLR(PF-PLR=1/0 = 0.006, HR=36.89, 95% CI=2.82-8.99; PF-PLR=2/0 = 0.004, HR=240.49, 95% CI=5.50-10.69)were independent prognostic factors influencing patient survival (**Table 2**).

## Discussion

Lung cancer is the most prevalent malignant tumor globally and the leading cause of cancer-related deaths among individuals aged over 50 years. Approximately 85% of all lung cancer cases are attributed to non-small cell lung cancer (NSCLC) (1). Surgery remains the preferred treatment modality for stage I-IIIA NSCLC. However, there is significant variability in the 5-year postoperative survival rates among patients (20). The neutrophil-to-lymphocyte ratio (NLR), platelet-to-lymphocyte ratio (PLR), and the hemoglobin, albumin, lymphocyte, and platelet score (HALP) have the potential to function as predictors of prognosis for NSCLC patients following surgery (21–23). Recently, combined indexes such as the combination of plasma fibrinogen and neutrophil-to-lymphocyte ratio (F-NLR), the combination of platelet count and lymphocyte-to-monocyte ratio (COP-LMR), and the combination of platelet count and mean platelet volume (COP-MPV) have been utilized to assess the prognosis of patients (20, 24, 25). All of these combined indexes have demonstrated better predictive capabilities compared to individual indexes. We endeavored to utilize the combination of fibrinogen and platelet-to-lymphocyte ratio (F-PLR) to assess the postoperative prognosis of patients with NSCLC. To our knowledge, this study is the first of its kind to examine the value of the F-PLR score in predicting the prognosis of NSCLC patients.

In this study, we employed the receiver operating characteristic (ROC) curve to ascertain the optimal cut-off values for fibrinogen and F-PLR. The optimal cut-off value for fibrinogen was 3.90 g/L, and that for F-PLR was 213.2. After that, based on the cut-off values of fibrinogen and F-PLR, the patients were divided into three groups. Univariate analysis identified six indicators associated with the postoperative overall survival (OS) of patients with NSCLC. Subsequently, these indicators were incorporated into a multivariate analysis. The results of the multivariate analysis demonstrated that the utilization of adjuvant therapy after surgery, pathological stage, and F-PLR score were independent risk factors influencing the postoperative OS of NSCLC patients. Based on the above mentioned findings, we conclude that the F-PLR score stands as an independent prognostic risk factor for OS among patients diagnosed with NSCLC. We also separately evaluated the prognostic value of the F-PLR score in patients with stage I, II, and IIIA NSCLC. The results demonstrated that the F-PLR score was significantly correlated with OS in these NSCLC patients at stage I, II, and IIIA.

Numerous relevant studies have provided evidence that procoagulant and fibrinolytic factors are overexpressed within tumor tissues (26). Plasma fibrinogen, a protein closely associated with coagulation, participates in tumor-cell angiogenesis, proliferation, and metastasis through directly binding to tumor cells (10). Sheng, L. et al. (27) assessed the preoperative fibrinogen levels in 567 patients with NSCLC who underwent surgical treatment. Their findings revealed that, compared with patients having normal fibrinogen levels, those with elevated fibrinogen levels exhibited poorer progression - free survival (PFS) and OS. This finding is in accordance with the results of our study. Fibrinogen exerts a crucial function within the coagulation cascade, a physiological process that is essential for tumor progression and metastasis (28). Fibrinogen is intricately involved in the interaction between the tumor and the cellular stroma. It serves as a scaffold for tumor-associated growth factors, including vascular endothelial growth factor(VEGF) and fibroblast growth factor-2(FGF-2). This scaffolding function enables the facilitation of a microenvironment conducive to tumor cell proliferation and metastasis, thereby promoting the malignant progression of tumors (29, 30). Furthermore, fibrinogen has the ability to augment the interaction between tumor cells and platelets. Simultaneously, it promotes the activation of thrombin. Thrombin, in turn, is capable of converting fibrinogen into fibrin. This conversion leads to the formation of a stable framework and extracellular matrix surrounding the tumor cells. As a result, tumor cells are shielded from being eradicated by the body’s innate immune cells (31, 32).

Accumulating studies have demonstrated that approximately 10-57% of patients diagnosed with malignant tumors exhibit thrombocytosis (33). Platelet activation represents a crucial biological process that significantly contributes to cell carcinogenesis and tumor metastasis (34, 35). It is widely considered to be correlated with an unfavorable prognosis in patients afflicted with NSCLC. Mean platelet volume (MPV) is regarded as a biomarker for platelet activation (36). Gao, L. et al. (25) investigated the association between the combined preoperative platelet count and mean platelet volume (COP-MPV) scores and the prognosis of 546 patients with NSCLC who underwent surgical treatment. The study results indicated that patients with lower COP-MPV scores exhibited more favorable OS and DFS compared to those with higher scores. Platelets contribute significantly to tumor progression through the release of various cell growth factors, including VEGF, platelet-derived growth factor (PDGF), transforming growth factor-β (TGF-β), and fibroblast growth factor (FGF) (37, 38). Platelet-derived transforming growth factor (TGF) promotes epithelial- mesenchymal transition by activating the Smad and NF-κB signaling pathways, thereby enhancing the metastatic potential of tumor cells (39). Furthermore, tumor cells can impact tumor cell proliferation and induce the differentiation of megakaryocytes in the bone marrow towards platelets. This is achieved through the production of platelet - promoting cytokines and interleukin-6 (40). Tumor cells are also capable of evading the immune system by inducing platelet aggregation. This aggregation mechanism enables tumor cells to elude immune surveillance (41). Lymphocytes assume a crucial role in the antitumor immune response, primarily by exerting inhibitory effects on tumor cell growth (42). Lymphopenia is an independent prognostic factor for both OS and DFS in patients with cancer. Moreover, it is highly prevalent among patients with advanced-stage cancer (43). A growing body of studies has revealed that the platelet-fibrin (progenitor) axis can contribute to tumor metastasis by obstructing the elimination of tumor cells carried out by the body’s natural killer cells (44). Moreover, platelets and fibrinogen collaborate to drive the processes of tumor cell growth, invasion, and hematogenous metastasis. They achieve this by promoting tumor-associated angiogenesis and augmenting the ability of tumor cells to adhere to the surrounding tissues. This cooperative action between platelets and fibrinogen contributes significantly to the malignant progression of tumors, highlighting their importance in cancer pathophysiology (39, 45, 46). Huang, J. et al. (18) incorporated fibrinogen and the platelet-to-lymphocyte ratio in the prognostic analysis of patients with limited upper uroepithelial carcinoma. The findings of our study showed that the F-PLR score measured before surgery could independently predict the survival outcomes of these patients. Specifically, the higher the F-PLR, the poorer the cancer-specific survival (CSS) and OS of the patients. Yang, Y. et al. applied the F-PLR to predict the prognosis of patients with esophageal squamous carcinoma. The results demonstrated that the F-PLR score was closely associated with the OS of these patients, indicating its potential utility as a tool for prognostic prediction in esophageal squamous carcinoma patients (19). The results indicate that the F-PLR score can serve as a tool for predicting the prognosis of patients with malignant tumors, and it demonstrates promising prospects for applications, potentially spanning across multiple aspects of cancer management, such as personalized treatment planning and long-term outcome assessment.

Although our study is the first to utilize the F-PLR score for determining the prognosis of patients with NSCLC who undergo surgical treatment, several limitations are present in our research. First, this study was conducted at a single center in China. Although our cohort is representative of the typical patient population in this region, the homogeneity in ethnic background and local treatment protocols may limit the generalizability of our findings to other geographic and healthcare settings. Therefore, future multinational, multi-center studies are warranted to validate our results in more diverse populations.

In conclusion, the preoperative F-PLR scores of patients can serve as an independent prognostic factor for NSCLC patients. Moreover, these scores can be utilized to preoperatively categorize these patients into three distinct groups. The F-PLR score has the potential to assist clinicians in preoperatively identifying high-risk patients and formulating personalized treatment strategies tailored to the specific needs of each patient.

## Data Availability

The original contributions presented in the study are included in the article/**Supplementary Material**. Further inquiries can be directed to the corresponding authors.
